# Yellow Fever Virus Infectivity for Bolivian *Aedes aegypti* Mosquitoes

**DOI:** 10.3201/eid1009.031124

**Published:** 2004-09

**Authors:** John-Paul Mutebi, Alberto Gianella, Amelia Travassos da Rosa, Robert B. Tesh, Alan D. T. Barrett, Stephen Higgs

**Affiliations:** * Chicago Department of Public Health, Chicago, Illinois, USA;; † CENETROP, Santa Cruz, Bolivia;; ‡ University of Texas Medical Branch, Galveston, Texas, USA

**Keywords:** yellow fever virus, *Aedes aegypti*, mosquito, arbovirus, vector-borne disease, dispatch

## Abstract

The absence of urban yellow fever virus (YFV) in Bolivian cities has been attributed to the lack of competent urban mosquito vectors. Experiments with *Aedes aegypti* from Santa Cruz, Bolivia, demonstrated infection (100%), dissemination (20%), and transmission of a Bolivian YFV strain (CENETROP-322).

Yellow fever virus (YFV) may cause severe hemorrhagic fever in humans. The virus is transmitted between susceptible vertebrate hosts by infected mosquitoes in the genera *Aedes*, *Haemagogus*, or *Sabethes* (*1*). In the Americas, YFV occurs in two transmission cycles. In the jungle/sylvatic cycle, the virus is transmitted between susceptible monkeys, and possibly other vertebrates, by tree-hole–breeding mosquitoes (*1*). Jungle yellow fever (YF) cases occur when these infected vectors feed on susceptible humans. In the urban cycle, YFV is transmitted to humans by *Aedes aegypti* mosquitoes (*1*). In 2003, a total of 226 cases of jungle YF were reported from South America to the Pan American Health Organization, and as of June 23, ongoing outbreaks in Bolivia, Brazil, Colombia, and Peru during 2004 have thus far resulted in 86 confirmed cases and 41 deaths (*2*).

An *Ae. aegypti* eradication campaign initiated by the Pan American Sanitary Bureau in 1947 eliminated this species from most of Central and South America, and urban YF disappeared from the Americas in the 1940s. However, during the past 20 years, many countries abandoned *Ae. aegypti* control measures, and this urban vector now reoccupies almost the entire area of its distribution preeradication (*1*). *Ae. aegypti* was eradicated from Bolivia during the 1960s and 1970s but reappeared in the city of Santa Cruz in 1980, and epidemics of dengue fever occurred during the 1980s and 1990s (*3*). In 1997 to 1998, six cases of YF were reported among Santa Cruz residents, and some were regarded as urban YF cases (*3*); despite a population >1 million, low vaccine immunization coverage, and the presence of *Ae. aegypti* (*3*), no urban YF outbreak occurred. Based on these observations, researchers have suggested that sylvan strains of YFV circulating in Bolivia may not be infective for Bolivian *Ae. aegypti*. This study examined that hypothesis and the infectivity of a Bolivian strain of YFV for Bolivian *Ae. aegypti*.

## The Study

All work involving infectious YFV was performed in biosafety level 3 facilities at the University of Texas Medical Branch. Three human isolates of YFV were used: CENETROP-322 (La Paz Department, Bolivia, 1999), Jimenez (Panama, 1974), and Asibi (Ghana, 1927). To facilitate transmission from a viremic vertebrate, viruses were adapted by serial passage through Syrian golden hamsters (*Mesocricetus auratus*), following the model of Tesh et al. (*4**,**5*); CENETROP-322 and Jimenez were passaged 11 times, and the Asibi strain was passaged 10 times.

The SC strain of *Ae. aegypti* was started with mosquitoes collected from Santa Cruz, Bolivia, in 2001. Mosquitoes used in this experiment were from laboratory-reared F2-F3 generation. The REX-D strain, an old laboratory colony originally started with mosquitoes collected in Rexville, Puerto Rico and of previously defined susceptibility to YFV infection (*6*) was used as a control. Mosquitoes were maintained as previously described (*7*).

Three hamsters were injected intraperitoneally (IP) with 100 µL of clarified liver homogenate, which contained approximately 10^6^ log_10_ tissue culture infectious dose 50% (TCID_50_/mL) of each YFV strain. Three days after infection, when viremia levels have been shown to peak (*4*), hamsters were anesthetized (50 mg *Pentobarb*/kg IP) and simultaneously exposed to 10-day-old *Ae. aegypti* SC or REX-D mosquitoes for 1 h. Fully engorged mosquitoes in each group were placed in separate cages and incubated for 15 days at 28°C and 80% relative humidity on a diet of 10% sucrose. Hamster blood samples were collected immediately afterward and stored at –80°C for viral assay.

### Virus Transmission

At day 15 after infection, mosquitoes were allowed to feed on 8-day-old mice. (Mice were used in preference to adult hamsters because they are more susceptible to fatal infection.) After feeding, mosquitoes were assayed for YFV infection and dissemination by whole-body titration and immunofluorescence assay (IFA) of head-squash material, respectively (*7*). For IFA, a broadly reactive antiflavivirus monoclonal antibody (813) with biotin-streptavidin amplification was used (*8*). Suckling mice were observed for illness and death. The brains of two paralyzed mice were tested for viral antigen by culturing on Vero cells. At day 14 after exposure, serum specimens from surviving mice were tested for anti-YFV antibodies by hemagglutination inhibition (HI) test (*9*).

All three strains of YFV caused viremia in hamsters on day 3 after infection, with titers of 8.5, 8.7, and 7.3 TCID_50_log_10_/mL (CENETROP-322, Jimenez, and Asibi, respectively), as determined by assay in mosquito cell cultures (*4*). Determination of mosquito infection and dissemination rates showed that all YFV strains were able to infect both strains of *Ae. aegypti*, although infection rates varied from 15.1% to 100%. Mean total mosquito YFV titers were relatively low, but the presence of virus after 15 days, evidence of dissemination in the insect, and transmission data are all indicative of replication. At day 15 postinfection, 100% of SC *Ae. aegypti* were infected with CENETROP-322. Infection rates and mean viral titers for CENETROP-322 were higher in SC *Ae. aegypti* than in the REX-D strain (Table 1). Infection rates of CENETROP-322 and Jimenez were higher than the rate for Asibi in both mosquito strains.

**Table 1:** Infection, dissemination, and virus titers for three strains of yellow fever virus, CENETROP-322, Jimenez, and Asibi, in two strains of *Aedes aegypti* mosquitoes, Santa Cruz and REX-D, at day 15 postinfection^a^

*A. aegypti*	Virus strain	Hamster serum titer (TCID_50_ log_10_/mL)	No. infected by titration (5%)	Mean titer of positives (TCID_50_ log_10_/mL	Dissemination rate by IFA (%)
Santa Cruz	CENETROP-322	8.5	10/10 (100)	3.5	20
Santa Cruz	Jimenez	8.7	29/31 (93.5)	3.5	34.5
Santa Cruz	Asibi	7.3	3/26 (15.1)	3.5	0
REX-D	CENETROP-322	8.5	19/30 (63.3)	1.5	80.7
REX-D	Jimenez	8.7	27/30 (90)	3.5	73.4
REX-D	Asibi	7.3	13/30 (43.3)	4.0	38.4

^a^TCID_50_, tissue culture infectious dose 50%; IFA, immunofluorescence assay.

Virus titers in the mosquitoes varied considerably but were lowest in REX-D strain insects infected with CENETROP-322 (Table 1). These mosquitoes had the highest dissemination rates (80.7%), which indicates little correlation between virus titer and dissemination rates. Dissemination rates were highest in the REX-D strain; but our data demonstrate that both Panamanian and Bolivian strains of YFV disseminated in Santa Cruz *Ae. aegypti* (Table 1).

Transmission trials used 8-day-old mice to feed mosquitoes that had ingested YFV 15 days earlier (Table 2). Five mice were used per virus strain. The reluctance of mosquitoes to feed on suckling mice precluded an evaluation of all YFV-mosquito combinations. However, HI results indicated that antibodies against YFV (320 titer, Table 2) developed in one mouse exposed to SC mosquitoes infected with CENETROP-322, which indicated transmission by the Bolivian *Ae. aegypti*. In addition, Jimenez and Asibi strains of YFV were transmitted by the REX-D mosquitoes. Transmission was confirmed by recovering YFV by culture from dead mice.

**Table 2:** Transmission of yellow fever virus by infected *Aedes aegypti* mosquitoes to suckling mice^a^

*Ae. aegypti* strain	Virus strain	No. mice infected/ No. exposed (%)	HI titer for 4 U of antigen		
			YFV	SLEV	WNV
Santa Cruz-	CENETROP-322	1/5 (20)^b^	0	0	0
			0	0	0
			320	0	0
			0	0	0
			0	0	0
REX-D	Jimenez	4/5 (80)^c^	NT	NT	NT
			NT	NT	NT
			NT	NT	NT
			NT	NT	NT
			NT	NT	NT
REX-D	Asibi	3/5 (60)^b^	160	0	0
			0	0	0
			0	0	0
			80	0	0
			40	0	0

^a^HI, hemagluttination-inhibition; YFV, yellow fever virus; SLEV, St. Louis encephalitis virus; WNV, West Nile virus; NT, not tested. ^b^Infection determined by presence of anti-YF antibodies in mice sera (HI test). 0 indicates a titer of <1:20. ^c^Infection determined by death/virus detection for suckling mice.

## Conclusions

Susceptibility to YFV infection is highly variable in mosquitoes from different locations (*10**–**12*) and may be influenced by selection (*6*) and colonization (*13*). Although the use of Bolivian mosquitoes with few laboratory-reared generations compromised our ability to use large numbers, obtaining competence data as possibly representative of wild, noncolonized, mosquitoes was important. Dissemination and transmission by Santa Cruz *Ae. aegypti* indicate their ability to serve as vectors for a Bolivian strain of YFV. A critical component of this study was the use of a hamster model for YFV (*4*). The high viremia levels in hamsters (*4**,**5*) facilitate oral infection of mosquitoes and more closely resemble natural infection than feeding the insects on artificial blood meals. Suckling mice remain useful because of their sensitivity to YFV infection.

We could argue that by passing the virus in hamsters, the virus phenotype may be altered with respect to vector infectivity. However, after equivalent passages, the infectivity of the Bolivian, Panamanian, and African strains differed. The Jimenez strain was highly infectious for Bolivian *Ae. aegypti* (93.5%), with a relatively high dissemination rate (34.5%). In contrast, the Asibi was relatively noninfectious for the Bolivian mosquitoes. Considering the numbers of mosquitoes and virus strains involved, we cannot conclude that this finding reflects a general trend of incompatibility between South American *Ae. aegypti* and YFV of African origin. However, the results obtained are in close agreement with the findings by Tabachnick et al. (*12*). Johnson et al. (*14*), using Brazilian strains of *Ae. aegypti* and YFV, reported similar results of 35% infection rates and 25% dissemination rates. Lourenço-de-Oliveira et al. (*11**,**15*) observed infection rates from 0% to 48.6% in Brazilian *Ae. aegypti* infected with Brazilian YFV. In comparison, we found higher infection rates for Panamian and Bolivian YF viruses (63.3%–100%), but this finding may reflect our use of a viremic animal to infect the mosquitoes, whereas Tabachnick et al. (*12*) and Johnson et al. (*14*) used artificially prepared blood meals. Our results also demonstrate that passaging YFV in hamsters does not compromise the ability of the virus to infect mosquitoes and that the hamster model is useful to study mosquito competence for YFV.

In conclusion, our results do not support the hypothesis that Bolivian strains of YFV cannot infect Bolivian *Ae. aegypti* and demonstrate that the recolonizing (after 1980) South American strains of *Ae. aegypti* are potential YFV vectors. The reason urban YF epidemics have not yet occurred in South America, including in the city of Santa Cruz, Bolivia, where some cases were recently reported within the city limits (*3*), is still unknown. The mosquito infection rates observed in our study were higher that those reported by Lourenço-de-Oliviera et al. (*11*), and we also demonstrated YFV transmission (albeit at a low level). Thus, if YFV were to be reintroduced into urban areas of South America, a transmission cycle could possibly be established. The absence of epidemic YF may be the result of other factors, including widespread deforestation and less opportunity for YFV to move out of the sylvatic cycle, better mosquito control, the local population's YFV vaccine status, and, possibly, heterologous antibodies to other flaviviruses such as dengue (*5*).
